# Tyrosyl-DNA Phosphodiesterase I N-Terminal Domain Modifications and Interactions Regulate Cellular Function

**DOI:** 10.3390/genes10110897

**Published:** 2019-11-06

**Authors:** Evan J. Brettrager, Isaac A. Segura, Robert C. A. M. van Waardenburg

**Affiliations:** Department of Pharmacology and Toxicology, University of Alabama at Birmingham, Birmingham, AL 35294-0019, USA; evanb@uab.edu (E.J.B.); is325@uab.edu (I.A.S.)

**Keywords:** Tdp1, DNA topoisomerases, DNA-adducts, DNA metabolism, post-translational modifications, protein–protein interactions, catalytic mechanism

## Abstract

The conserved eukaryotic DNA repair enzyme Tyrosyl-DNA phosphodiesterase I (Tdp1) removes a diverse array of adducts from the end of DNA strand breaks. Tdp1 specifically catalyzes the hydrolysis of phosphodiester linked DNA-adducts. These DNA lesions range from damaged nucleotides to peptide-DNA adducts to protein-DNA covalent complexes and are products of endogenously or exogenously induced insults or simply failed reaction products. These adducts include DNA inserted ribonucleotides and non-conventional nucleotides, as well as covalent reaction intermediates of DNA topoisomerases with DNA and a Tdp1-DNA adduct in trans. This implies that Tdp1 plays a role in maintaining genome stability and cellular homeostasis. Dysregulation of Tdp1 protein levels or catalysis shifts the equilibrium to genome instability and is associated with driving human pathologies such as cancer and neurodegeneration. In this review, we highlight the function of the N-terminal domain of Tdp1. This domain is understudied, structurally unresolved, and the least conserved in amino acid sequence and length compared to the rest of the enzyme. However, over time it emerged that the N-terminal domain was post-translationally modified by, among others, phosphorylation, SUMOylation, and Ubiquitinoylation, which regulate Tdp1 protein interactions with other DNA repair associated proteins, cellular localization, and Tdp1 protein stability.

## 1. Introduction

**Figure 1:**
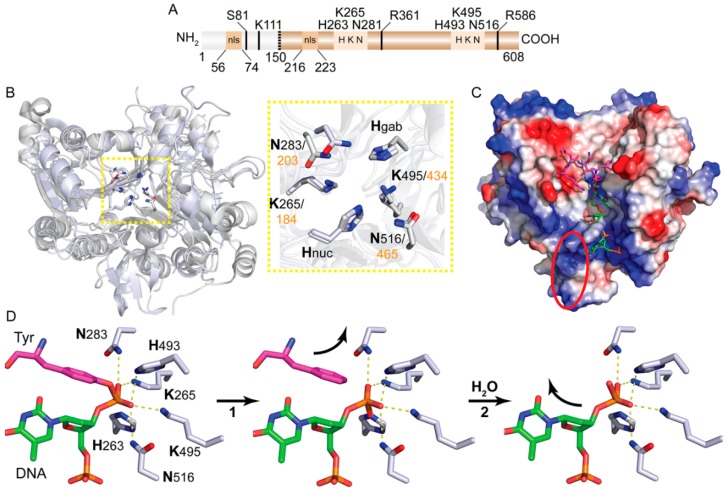
Tyrosyl-DNA phosphodiesterase I (Tdp1) interaction and catalysis of adducted DNA. (**A**) Linear representation of human Tdp1 domain structure. The structurally unresolved N-terminal domain (amino acid 1–149) in gray and the structurally resolved catalytic domain (amino acid 150–608) in brown. Boxes show the location of the two nuclear localization signal (nls) and the two HKN-catalytic motifs with their residue numbers above. Post-translationally modified residues (black line) are shown: S81 is phosphorylated; K111 is SUMOylated; R361 and R586 are demethylated. (**B**) Carbon-backbone overlay in a cartoon representation of the resolved catalytic core domain structures of Human Tdp1 (blue, PDB:1NOP) [31] and yeast Tdp1 (gray, PDB:1Q32) [9]. Although the amino acid sequence homology is only 38% conserved (identical + similar residues), the structural conservation is remarkably high, as their catalytic residues are nearly superimposable (zoom), with some deviations occurring only at the periphery. (**C**) Surface electrostatic potential (estimation generated by MacPyMol) of human Tdp1. The ssDNA (green with DNA-phosphate backbone in orange) located in the positively charged DNA binding-gorge is covalently bonded to vanadate (yellow). The vanadate is also bond to the active site tyrosine of hTopo1 as part of the protease resistance hTopo1 peptide fragment positioned in the protein docking basin. This basin displays a more neutral charged bottom with neutral/negatively charged enclosed walls. In the red ellipse is the location where the non-adducted DNA strand interacts as revealed in the crystal structure by Flett et al. [32]. Electrostatic charge is represented in gradients from blue (positive) to white (neutral) to red (negative). (**D**) Tdp1 two step catalytic cycle represented from a structural perspective (adjusted from 1NOP structure shown above). The first HKN-motif provides Lys265 and Asn283 that stabilize the adducted phosphate group via formation of hydrogen-bonds, which is supported by hydrogen-bonds formed by Lys595 and Asn516 from the second HKN-motif. Lys595 and Asn 516 also function in an electron-relay mechanism to first provide a proton to the general acid base His493 (His^gab^) that maintains the nucleophilic His263 (His^nuc^) in its deprotonated phase [9,28,31,33]. After docking and stabilizing the adducted DNA strand, the nucleophilic His263 will hydrolyze the 3’phospho–tyrosyl bond by forming a 3’phospho–hystidyl linkage, covalently attaching Tdp1 to the end of the DNA (Step 1). This step releases Topo1 from the DNA after the general acid/base His493 donates its proton to the phenoxy anion of tyrosine to prevent reformation of the original DNA-adduct [34]. The now nucleophilic general acid/base His493 will activate a water molecule by accepting its proton, while the remaining hydroxyl will hydrolyze the Tdp1–DNA bond releasing Tdp1 from the DNA end. All structures were generated using MacPyMol (Molecular Graphics System, Schrödinger, LLC).

The eukaryotic DNA repair enzyme Tyrosyl-DNA phosphodiesterase I (Tdp1) was discovered by Howard Nash and co-workers as an enzyme activity that cleaves a 3’phospho–tyrosyl bond [1] and was subsequently isolated from *Saccharomyces cerevisiae* [2,3]. The 3’phospho–tyrosyl linkage is the hallmark chemical bond of Tyrosine-recombinases active-site tyrosine to the 3’ end of DNA. This includes enzymes such as Cre-recombinase from phage P1 and Flp-recombinase in *S. cerevisiae*, but also eukaryotic DNA topoisomerase I (Topo1) [4]. The *TDP1* gene is highly conserved among eukaryotes. Sequence analysis places Tdp1 within the phospholipase D (PLD) superfamily based on the presence of two conserved histidine-lysine-asparagine (HxKx_(n)_N, x being any amino acid)-catalytic motifs [5,6,7]. However, Tdp1 is placed in a distinct sub-class of the PLD superfamily as the *Tdp1-motif* lacks the aspartate residue (D) of the conserved *PLD-motif* (HxKx_4_Dx_6_N) [5]. Subsequent comparison of the crystal structures of human and yeast Tdp1 catalytic domains with the crystal structures of PLD-family member proteins confirmed Tdp1’s PLD-classification [8,9]. Moreover, this structural comparison highlighted that besides the virtually superimposable catalytic residues, the two α-β-α halves are structured in a pseudo-2-fold axis of symmetry, which is also a conserved feature between PLD-proteins and Tdp1. Each “α-β-α half” contributes a catalytic HK(D)N-motif to the catalytic pocket and together form the catalytic domain (reviewed in [10]). Compared to PLD-proteins, Tdp1-proteins contain an extra N-terminal domain. This domain remains structurally unresolved. However, its function is slowly being uncovered. Figure 1A shows a linear representation of the general human Tdp1 domain structure and the location of residues discussed in this review.

The existence of a “Tdp1-like activity” was speculated for decades by topoisomerase investigators. Yang et al. directly implied that this enzyme-activity might be involved with resistance to, or reduced activity of, DNA Topoisomerase-targeting chemotherapeutics [1]. Since its discovery, the variety of DNA-adduct substrates for Tdp1 has grown in number and diversity. Tdp1 substrates can be divided into two general classes: (**1**) Protein/peptide-DNA adducts including the original Topo1-DNA covalent complex (Topo1cc) or its protease resistant peptide-DNA adduct formed from Topo1 proteolysis, which are both popular model substrates to study Tdp1 function, mechanism, and substrate interactions, and (**2**) single damaged/modified nucleotides such as oxidatively damaged nucleotides as a result of bleomycin treatment, a favorite model for this kind of Tdp1 substrate [10,11,12]. 

Tdp1 is not essential, as *tdp1* knockout yeasts (*Saccharomyces cerevisiae* and *Schizosaccharomyces pombe*) and mice are viable, but in mice it shows a synthetic lethal interaction with ATM (Ataxia telangiectasia mutated) [3,9,13,14,15,16]. Tdp1 in cells is located in the DNA containing compartments, the nucleus/nucleolus and mitochondria, but also in the cytosol, which might function as a reservoir [17,18,19,20]. The broad array of DNA-adducts and the general cellular distribution suggest Tdp1 plays an important but redundant role in maintaining nuclear and mitochondrial DNA stability. Intriguingly, many cancer derived cell lines and tumor samples show elevated expression of Tdp1 ([20,21,22,23,24], unpublished observations). Moreover, increased Tdp1 levels are associated with genomic instability and sensitivity to chemotherapeutic induced DNA damage [9,20,21,25,26,27,28]. Consequently, healthy tissues/cells maintain Tdp1 protein levels at low, almost non-detectable levels ([20], unpublished observations). Herein, we will focus on the role of the understudied, structurally unresolved Tdp1 N-terminal domain, specifically its involvement with post-transcriptional modifications and Tdp1-protein/Tdp1-DNA adduct interactions.

## 2. Tdp1 Catalytic Mechanism

Tdp1 mediated catalysis utilizes two catalytic histidines and is centered on the formation of a transient covalent Tdp1-DNA reaction intermediate [2,3]. The chemistry of this catalysis is highly conserved between PLD and Tdp1 enzymes as was recently shown by quantum mechanical analysis [29,30]. The alignment of the resolved crystal structure of yeast and human Tdp1 catalytic domains displayed a high degree of conservation of the enzyme architecture with poorly conserved regions at the periphery (Figure 1B) [8,9,10]. The lack of a full-length structure of either yeast or human Tdp1 represents a challenging gap in our knowledge to fully comprehend the regulation of catalysis and interaction with the different DNA-adducts. The hTdp1-DNA-hTopo1 peptide fragment resolved crystal structure [31] revealed a narrow positively charged DNA binding-gorge that fits only single strand DNA. This ssDNA binding-gorge runs into the catalytic pocket that is on the opposite side connected to a wide basin where the protein/peptide fragment will dock (Figure 1C). This protein docking basin has a more neutrally charged bottom and its enclosed walls exhibit negatively charged patches (Figure 1C). A recently resolved crystal structure of two Tdp1 molecules (catalytic core only) bound to either end of a hybrid single/double stranded DNA-substrate gave a first impression of how the “non-adducted” strand might interact with Tdp1’s catalytic core [32]. 

This new structure showed that the non-adducted strand interacts with negative patches that are spread-out over Tdp1’s surface (red ellipse Figure 1C). The DNA binding-gorge seems to function as a “guide” to correctly position the DNA-adduct phosphodiester linkage between both HKN-motifs within the catalytic pocket (Figure 1C). This architecture of the *DNA binding-gorge-catalytic pocket-protein docking-basin* is highly conserved between yeast and human, with virtually superimposable positions of their catalytic HKN-motifs (zoom box in Figure 1B) [8,9,10]. After docking of the DNA-adduct, the Lys- and Asn-residues within two HKN-motifs “capture and stabilize” the phosphodiester linkage via hydrogen-bonds (yellow lines in Figure 1D). Simultaneously, the His-residues orchestrate hydrolysis of the phosphodiester bond via two coordinated S_N_2 nucleophilic attacks [1,5,9,28,34,35,36]. We will use the 3’phospho–tyrosyl linkage as a model substrate to describe the Tdp1 catalytic mechanism (Figure 1D). During step 1, or the formation step, the “nucleophilic” histidine (His^nuc^: Human His263 and yeast His182, Figure 1B) attacks the 3’phospho–tyrosyl linkage resulting in the formation of a 3’phospho–histidyl bond covalently attaching Tdp1 to the DNA end. In step 2, or the resolution step, the “general acid/base” histidine (His^gab^: His493 in human and His 432 in yeast, Figure 1B) facilitates Tdp1 detachment from the DNA end by activating a water molecule that hydrolyzes the 3’phospho–histidyl bond. During the transition of step 1 to step 2, reformation of the original Topo1-DNA adduct is prevented through protonation of the “leaving" tyrosine by the His^gab^ [34]. These coupled trans-esterification reactions that are utilized during Tdp1 catalysis obviate the necessity for divalent cations and ATP hydrolysis. Unlike DNA topoisomerases that dissociate from their transient covalent enzyme–DNA complexes via religation of the nicked DNA, Tdp1 does not mediate religation of the nicked DNA strand. Before DNA ligase can religate the nicked strand, polynucleotide kinase 3’ phosphatase (PNKP) in mammalian cells and the 3’ phosphatase Tpp1 and 5’ kinase Trl1 enzymes in budding yeast will facilitate “reversal” of the phosphate- and hydroxyl-end groups [37,38,39,40,41,42]. Thus, cells take a “calculated” risk by utilizing Tdp1 for the removal of DNA-adducts, given that Tdp1 forms itself a potentially toxic transient covalent enzyme–DNA reaction intermediate. 

## 3. Diversity of Tdp1 Substrates

Tdp1 is able to hydrolyze a broad array of phosphodiester linked 5’- and 3’-DNA adducts. These Tdp1 substrates can be divided into two classes: (**1**) Large DNA-adducts, such as protein–DNA and peptide–DNA adducts/crosslinks, and (**2**) small DNA-adducts consisting of damaged or non-canonical nucleotides. These DNA-adducts are generated by chemotherapeutic/exogenous agents (e.g., topotecan, etoposide, bleomycin, nucleoside analogs), irradiation, and endogenously generated molecules such as reactive oxygen species and RNA molecules inserted into DNA. This highlights a general role for Tdp1 in maintaining genome stability. Tdp1 functions as a limited exonuclease; it catalyzes the removal of *one* “nucleotide-adduct,” and its reaction product, a phosphoryl group, impedes further catalysis by Tdp1 [1,2,35]. However, Tdp1 has not been demonstrated to act as an endonuclease. 

The **first class** of *large DNA-adducts* includes enzyme-DNA adducts such as those formed by nuclear and mitochondrial Topo1. Topo1 and Topo1mt both form a 3’phospho–tyrosyl bond with nuclear DNA and mitochondrial DNA, respectively. The formation of this transient Topo1–DNA covalent complex (Topo1cc) allows for the unwinding of DNA supercoiling to relieve torsional stress before the 5’-hydroxyl end initiates religation of the nicked DNA strand [43,44,45]. This transient Topo1cc is the sole target of the camptothecin (CPT) class of chemotherapeutics that include the clinically active water-soluble CPT analogs, topotecan (TPT), and the prodrug irinotecan (CPT-11, with SN-38 as active metabolite) (reviewed in [46]). These agents reversibly intercalate into the enzyme-DNA cleavage site impeding religation of DNA-ends that prevents resolution of the Topo1–DNA bond [47,48,49,50]. Besides CPT or other Topo1cc targeting molecules, local DNA perturbations can stabilize Topo1ccs (reviewed in [10,11,46]). These DNA perturbations include: Incorporation of cytarabine (Ara-C) or other non-canonical nucleotides such as gemcitabine, DNA mismatches in the cleaved strand, abasic sites, 7,8-dihydro-8-oxoguanine (8-oxoG), and single strand nicks generated by endogenous ROS in the non-cleaved strand [51,52,53,54,55,56]. Furthermore, platinum-DNA adducts induced by chemotherapeutics such as *cis*platin (cDDP) and other platinum-analogs can trap Topo1ccs [57,58]. Tdp1’s role in the removal of the above discussed Topo1ccs in the cells can mediate drug resistance to pharmacologic inhibitors of Topo1. Accordingly, Tdp1 overexpression in Chinese Hamster Lung (CHL) cells and Human Embryonic Kidney (HEK293) cells results in increased resistance to CPT induced DNA damage [17,59]. On the other hand, deletion of *TDP1* in *Saccharomyces cerevisiae, Schizosaccharomyces pombe*, DT40 chicken cells, HEK293 cells, and mice enhances cell sensitivity to CPT [2,3,9,13,14,15,60,61,62,63]. Tdp1 is also critical for mitochondrial homeostasis by removing Topo1mtcc’s formed between Topo1mt and mtDNA [18,38]. The sensitivity of Topo1mt to the DNA perturbations discussed above for nuclear Topo1 is not well investigated, but we anticipate this to be similar, as indirectly suggested by the increased sensitivity of *tdp1^-/-^* mitochondria to reactive oxygen species induced DNA damage [38]. 

In addition to Topo1cc’s, Tdp1 is also able to resolve in trans a Tdp1–DNA reaction intermediate by hydrolyzing the 3’phospho–histidyl covalent linkage [9,36,64,65]. This was discovered upon the identification of a Tdp1 catalytic mutant—His^493^Arg—which forms the molecular basis for the rare autosomal recessive neurodegenerative disease spinocerebellar ataxia with axonal neuropathy, or SCAN1 [36,65]. This was corroborated via in vitro biochemical and in vivo/cell studies with the SCAN1-related His-Arg substitution and additional substitutions of either catalytic histidine [9,25,26,28,36,39]. The toxic phenotype induced by SCAN1 and related His^gab^ and His^nuc^ substitutions results from a longer half-life of the covalent Tdp1–DNA reaction intermediate (Figure 1D) [25,26]. The activity of the His^nuc^Ala was surprising, as it was reported to be catalytically inactive in vitro, yet when expressed as a full-length enzyme this mutant induces a Top1-dependent toxicity in yeast and human cell models. This mutant also showed catalytic activity in vitro, albeit dramatically reduced compared to wild type, and formed in vitro and in cells detectable Tdp1-DNA adducts [25,26]. The Tdp1 nucleophilic histidine is preceded by a highly conserved histidine residue that, in the case of a His^nuc^ to Ala mutation, can replace the nucleophilic catalytic function. Indeed, the double HH^nuc^ to AA mutant does not show catalytic activity and does not induce a toxic phenotype [25]. 

Tdp1 is also able to hydrolyze 5’-phosphodiester linkages such as the 5’phospho–tyrosyl bond. In eukaryotes, this phosphodiester bond covalently attaches the catalytic tyrosine of yeast DNA Topoisomerase II (Topo2), and in mammalian cells Topo2α and Topo2β isoforms to the 5’-end of DNA. This 5’phospho–tyrosyl linkage is also formed between DNA Topoisomerase III (Topo3) isoforms (one in yeast and two (α and β) in mammalian cells) and the 5’-end of DNA as reviewed in [10,11,45,46]. The ability to process 5’-phospho–tyrosyl bonds was first reported by Barthelmes et al. in HEK293 cells modified to overexpress Tdp1, which reduced the number of etoposide induced DNA strand breaks [17]. The podophyllotoxin derivative etoposide (VP16) reversibly stabilizes the Topo2cc by intercalating into the cleavage site, preventing religation [66,67]. Although originally reported not to be able to resolve 5’phospho–tyrosine linkages in vitro, subsequent studies in yeast and vertebrate cells showed that both yeast and human Tdp1 are able to hydrolyze a 5’phospho–tyrosine bond [1,17,63,68,69]. On the other hand, the Tdp1^-/-^ knockout mice did not show increased sensitivity to VP16. This suggests that higher eukaryotes exhibit multiple redundant DNA repair pathways that are able to resolve 5’-phospho–tyrosyl linked protein-DNA adducts (e.g., Topo2cc) [14,69,70,71]. Indeed, an additional enzyme activity called Tdp2 was identified in higher eukaryotic cells. Tdp2 displays a higher affinity for Topo2cc’s or 5’phospho–tyrosyl linkages than Tdp1 [72]. Like Tdp1, Tdp2 can resolve both 3’phospho- and 5’phospho-DNA adducts, but is structurally and enzymatically different [73,74]. Lower eukaryotic organisms, such as budding yeast, do not have a Tdp2 homolog and depend on Tdp1 catalysis to hydrolyze the 5’phosphodiester-linked DNA-adducts. 

The most recent addition to the Tdp1 substrate list is a PARP1 peptide-DNA adduct. One of PARP1-lysine residues is linked to the C1’atom of an apurinic/apyrimidinic (AP)-site as a result of a Schiff base reaction [75]. PARP1–DNA covalent linkages are formed via a Schiff base reaction during PAPR1 limited AP-Lyase activity. PARP1 is subsequently proteolyzed to a PARP1 peptide-Lys-DNA adduct which is removed, along with the damaged base it reacted with, by Tdp1 [75,76]. Dysregulation of the PARP1–DNA Schiff base reaction by PARP1 inhibitors forms the molecular basis that results in cytotoxicity [76,77,78]. These kinds of Schiff base mediated protein–DNA linked complexes are part of many catalytic mechanisms of DNA metabolizing proteins [79,80]. These Schiff base linked protein–DNA crosslinks/adducts are slowly coming to the forefront as potentially toxic endogenously generated lesions. Among these DNA metabolizing proteins are Ku80, Neil1 DNA glycosylase, and DNA polymerase β [79,81,82,83]. Thus, Tdp1 plays a critical role in maintaining genome stability and a healthy cell homeostasis by catalyzing the removal of trapped protein–DNA adducts/crosslinks.

The **second classification** of *small DNA adducts* is comprised of damaged nucleotides and inserted non-canonical nucleotides or ribonucleotides into the DNA. However, keep in mind that Tdp1 only removes these adducts from the end of nicked DNA strands. Small DNA adducts include: (1) AP- or abasic-sites and 3’phospho-glycolates (PGs) caused by endogenous Reactive Oxygen Species (ROS) or therapeutics such as bleomycin, or gamma-/UV-radiation; (2) alkylated nucleotides resulting from methyl methanesulfonate (MMS) or temozolomide treatment; and (3) non-canonical nucleotide/nucleoside analogs, such as the anticancer and antiviral drugs acyclovir (ACV), cytarabine (Ara-C), zalcitabine (ddC), sapacitabine (CNDAC), and zidovudine (AZT) [5,35,84,85,86,87,88,89,90,91]. However, DT40 tdp1-/- cells did not show an increased sensitivity to gemcitabine treatment [86]. Lebedeva et al. suggested that Tdp1 might be able to hydrolyze the phosphodiester bonds on the 3’- and 5’-side of a naturally generated AP-site (by incubation of ds-oligonucleotide that included a deoxy-Uracil nucleotide in the middle of the top strand with Uracil-DNA glycosylase to generate the AP site) [89]. Overall, this suggests that Tdp1 acts as a limited 3’-exonuclease by removing *one* single RNA or DNA nucleotide from the 3’-end of the DNA and as such generates a 3’-phosphoryl DNA-product that acts as a Tdp1-endonuclease “STOP-signal” [35]. 

## 4. N-terminus Domain as the “Social” Mediator for Tdp1

The N-terminal residues of Tdp1 display the lowest homology among Tdp1 orthologs in sequence and in length, are easily proteolyzed in cells, and are structurally unknown and functionally understudied. The yeast and human Tdp1 core domain residues are about 38% conserved (identical + similar) [3,5]. The N-terminal domain, on the other hand, is not only significantly different in its length, as the human domain is about twice as long as the yeast domain (150 vs. 80 amino acids), but also displays 10% lower conservation than the core domain [9]. Moreover, purification of full-length Tdp1 from bacteria to yeast and insect cells to mammalian cells is very tricky due to proteolytic removal of this domain. This results in purification and resolution of the crystal structure of the Tdp1 core domain by itself [8,9]. The early observation that full-length and N-terminally truncated (catalytic core) human Tdp1 exhibits similar single turnover activity did not stimulate functional studies. However, this does not exclude the significance of the N-terminal domain for Tdp1 function in cells. Indeed, there is a slow but increasing accumulation of reports that present that the N-terminal residues of human Tdp1 are post-translationally modified, including phosphorylation and SUMOylation. Moreover, this domain also facilitates Tdp1–protein interactions with other DNA damage response and repair proteins (Table 1). Tdp1 post-translational modifications do affect specific Tdp1–protein interactions in a spatial and temporal manner. This suggests that within the cellular environment, these modifications and/or protein interactions might function to guide Tdp1 to the correct cellular compartment and/or to the appropriate DNA-adducts.

### 4.1. Phosphorylation

Zhou et al. were the first to describe the post-translational modification—phosphorylation—of nuclear and cytosolic Tdp1 in response to irradiation [91]. Moreover, they determined this was a Ser/Thr related modification, as protein phosphatase I treatment removed the phosphor-modification while the tyrosine specific YOP phosphatase had no effect. Subsequently, Das et al. detected that Tdp1 was phosphorylated in response to CPT treatment at Ser81 as a result of Ataxia telangiectasia mutated (ATM) and/or DNA-dependent protein kinase (DNA-PK) activity [92]. hTdp1S^81^-phosphorylation is within the N-terminal domain, which affects Tdp1 stability and stimulates interaction with the Base Excision Repair (BER) scaffold protein XRCC1 (X-ray repair cross-complementing protein 1), resulting in Tdp1 recruitment to the site of DNA damage [38,92]. In addition, this modification also enhanced the interaction between Tdp1 and DNA ligase IIIα (Lig3α), the final enzyme of BER and alternative non-homologous end joining (altNHEJ), to obtain an intact DNA strand [38]. These observations showed the dynamics involved in regulating Tdp1 interaction with XRCC1 and Lig3α, in which the N-terminal residues play a major role [39,93]. Indirectly, these observations support a role for Tdp1 in repair of mitochondrial DNA, as Lig3α is the mitochondria DNA ligase [18,38,99]. 

Conversely, Tdp1 interaction with NHEJ protein XLF/Cernunnos seems to be prevented by phosphorylation of Tdp1S^81^ [61]. XLF was shown to stimulate Tdp1 binding to dsDNA over ssDNA by forming a Tdp1/XLF/dsDNA complex [94]. The formation of this complex is dependent on XLF’s ability to bind DNA. Furthermore, Tdp1 interacts with the Ku70/80 complex that stimulates binding to DNA, and the interaction with DNA–PKcs results in a stimulation of the kinase activity of this complex which is dependent on the presence of Tdp1’s N-terminal domain [94]. This is consistent with the observation that Tdp1 plays an important role in NHEJ in yeast and mammalian cells [94,100]. Moreover, ectopic expression of the N-terminal truncated hTdp1 enzyme in HEK293tdp1^-/-^ cells did restore NHEJ to near wild type levels, consistent with their previous observation that XLF interacts with the Tdp1 core domain [94]. This observation suggests that the Tdp1/XLF/DNA interaction supersedes the Tdp1/Ku70/80 N-terminal domain dependent interaction, rendering the question “*Does the Tdp1/XLF/DNA complex stimulate DNA–PK (complex of Ku70/70/DNAPKcs) binding?”.* Additionally, expression of the Tdp1S^81^A, a non-phosphorylatable mutant, only restored NHEJ activity to ~60% of wild type levels. The phospho-mimetic mutant Tdp1S^81^E did not affect NHEJ in tdp1^-/-^ knockout cells, but did restore resistance to CPT in these cells [61]. Thus, in the case of Tdp1 interaction with XLF, phosphorylation of Tdp1 prevents interaction of XLF and in extension seems to impair NHEJ.

### 4.2. PARylation

Tdp1 and PARP1 have a multi-faceted relationship: From protein–protein interactions with or without the formation of a multi-protein complex, to PARP1 catalyzed PARylation of Tdp1 and Tdp1 catalyzed removal of failed PARP1–DNA complex formation (as discussed in Tdp1 substrate section). As early as when Tdp1 was reported to interact with the BER scaffold protein XRCC1, it was also proposed to be in complex with polβ, polynucleotide kinase phosphatase (PNKP), Lig3, and PARP1 [39,89,93]. The epistatic relation of PARP1 and Tdp1 in the repair of topotecan/irinotecan stabilized Topo1cc’s was highlighted in Rhabdomyosarcoma cells treated with PARP1-inhibitor and Tdp1 knockdown, and DT40tdp1-/- cells treated with combinations of PARP1-inhibitor and CPT [20,85,95]. Moreover, Das et al. demonstrated that the C-terminus of PARP1 directly interacts with the N-terminal domain of Tdp1, which is stimulated by DNA and nicotinamide adenine dinucleotide (NAD^+^) [95]. PARP1 also PARylates Tdp1 at unknown lysine residues, which does not affect Tdp1 activity [95,101]. Moreover, PAR-modifications at DNA damage sites stimulate Tdp1 recruitment to laser-induced DNA damage, which might be dependent on Tdp1 interaction with XRCC1. XRCC1 contains a BRCT-domain that recognizes PAR-chains at DNA damage sites [95,101]. PARP1 also interacts with and PARylates Topo1, which has been shown to regulate Topo1 nuclear dynamics—transport of Topo1 from the nucleolus to the nucleoplasm—upon CPT treatment [102]. This observation provides a molecular signal for the previously observed translocation of Topo1 and potentially for the observed translocation of Tdp1 [17,102]. The close and intriguing relation between Tdp1 and PARP1 was also highlighted by the identification of PARP1 inhibitors from a cell-based Tdp1 targeted drug-screen [103].

### 4.3. SUMOylation

Human Tdp1 was also found to be SUMOylated at Lys111 within its N-terminal domain, which directly implies that Tdp1 interacts with Ubc9 (the sole SUMO E2 conjugation enzyme) [98]. The question here remains: “Is Tdp1 SUMOylation the result of the sole action of a direct interaction with Ubc9?” or “Is this a result of a Ubc9-E3 SUMO ligase mediated SUMOylation event?” Tdp1 was found to be SUMO modified with all three major SUMO isoforms (SUMO-1, -2, -3). Although the overall influence of Tdp1 SUMOylation on Tdp1–protein interactions or function is unclear, it was shown that SUMO modification does not affect Tdp1 in vitro activity. Moreover, SUMO modification of Tdp1 stimulates Tdp1 translocation to DNA damage sites, hence the Tdp1K^111^R SUMO-mutant has a reduced rate of single strand break repair in response to CPT treatment [95,98]. As with most SUMO modified proteins, only a small fraction of the Tdp1 protein pool is SUMOylated in cells [98], suggesting that this modification facilitates a rapid response to acute toxic DNA-adducts, such as CPT stabilized Topo1cc.

### 4.4. Ubiquitylation/Deubiquitylation

UCHL3 catalyzes deubiquitylation of ubiquitylated Tdp1 to prevent Tdp1 proteolysis in cells [96]. This suggests that Tdp1 is ubiquitylated by an unknown E2 ubiquitin conjugation/E3 ubiquitin ligase couple at unknown positions in Tdp1. Knockdown of UCHL3 results in lower levels of Tdp1 which increases cell sensitivity to CPT. Furthermore, Liao et al. found a correlation between UCHL3 levels and Tdp1 levels in cancer cells and lymphoblastoid cell lines derived from SCAN1 patient cells [96]. They concluded that UCHL3 regulates physiological Tdp1 enzyme levels, with low UCHL3/Tdp1 levels to be associated with neurodegeneration and elevated levels of UCHL3/Tdp1 to be associated with cancer [96]. It is currently unclear if this ubiquitylation of Tdp1 is associated with the formation of Tdp1-DNA adducts and if post-translational modification of Topo1cc’s with SUMO and/or Ubiquitin is involved. 

### 4.5. Methylation

Protein arginine methyltransferase PRMT5, but not PRMT9, catalyzes dimethylation of Tdp1 at Arg361 and Arg586, which are located in the core domain of Tdp1 [97]. PRMT5 and PRMT9 are type II protein arginine methyltransferases that catalyze symmetric arginine-guanidino methylation. Tdp1 dimethylation stimulates XRCC1 interaction and the removal of Topo1cc’s in response to CPT treatment. Knockdown of PRMT5 resulted in an increase of PAR- and γH2AX-levels in response to CPT treatment, which the authors related to the role of PRMT5 in Tdp1 catalyzed Topo1cc hydrolysis. Moreover, protein methylation regulates a general response to DNA damage via the modification of proteins such as histone H2A, H2B, H3, and H4, TOPO3β, TP53, MRE11, BRCA1, and many more DNA metabolizing proteins [104]. For example, PRMT5 inhibition affects homologous recombination activity, which is associated with increased sensitivity to, among others, topotecan, bleomycin, doxorubicin, cyclophosphamide, and many more DNA damaging agents [105].

## 5. In Summary

Over the last two decades, we have obtained some insight into the N-terminal domain of Tdp1, which is poorly conserved in length and amino acid sequence, is structurally unresolved and understudied. This N-terminal domain mediates many Tdp1–protein interactions that are often regulated by post-translational modification of N-terminal residues. Although the studies summarized herein only reflect a simplified view of Tdp1 interactions with proteins in the cellular environment, they emphasize that we have much to learn about Tdp1 spatial and temporal cellular function. Tdp1 regulation of recruitment to—and mechanics of interaction with—protein-DNA adducts, such as Topo1cc, are still understudied and mostly hypothetical. To improve our knowledge, we need structural, biochemical, and cell-based information of the Tdp1 holoenzyme with and without adducted DNA, including protein-DNA intermediates. Moreover, we need to obtain mechanistic insight into Tdp1 in trans N-terminal and catalytic core domain interactions and how these interactions regulate Tdp1 catalysis and substrate selection. For example, we have no comprehension of how Tdp1 gains access to the 3’phospho–tyrosyl bond, which is protected within the Topo1cc. However, Tdp1 in concert with ATM is essential for developing neuronal cell viability during embryogenesis, when these cells specifically accumulate toxic Topo1cc levels. Why would these rapidly dividing and high energy demanding cells proteolyze Topo1 of every Topo1cc before Tdp1 catalyzed hydrolysis? Tdp1 is able to remove Topo1 from Topo1cc in cells. Hence, the assumption that Tdp1 is not able to resolve a Topo1cc is extrapolated from in vitro experiments using truncated Tdp1 and/or truncated Topo1 enzymes, which does not recapitulate cellular conditions. 

Since the reported discovery of Tdp1 by the late Howard Nash in 1996 [1], we have gained fundamental understanding of Tdp1: Chemistry, tertiary structure of its catalytic core domain, and ability to resolve a diverse array of DNA adducts ranging from damaged nucleotides to peptide- and protein-DNA adducts. Although Tdp1 is a non-essential enzyme, it plays an important role in maintaining cellular homeostasis, while dysregulation of Tdp1 enzyme levels or catalysis can drive human pathology such as neurodegeneration and cancer. Conversely, Tdp1 also provides prospective treatment options through chemical catalytic inhibition or poisoning, which are currently under development to become potential therapeutics. 

## Figures and Tables

**Table 1:** Tdp1 post-translational modification and protein interaction partners.

Protein	Tdp1 domain	Response to	PTM^A^	Effector	Reference
ATM	N	CPT/IR	S^81^P		[92]
DNA–PK	N	CPT/IR	S^81^P		[92]
XRCC1	N	CPT/IR		↑S^81^P ^B^	[39,92,93]
Lig3α	N	CPT/IR		↑S^81^P ^B^	[38,39]
XLF	Core			↓S^81^P ^C^	[61,94]
Ku70/80	N				[94]
Ku70/80/DNAPkcs^D^	N		S^81^P		[94]
PARP1	N		K^?^PAR ^E^		[20,85,95]
UCHL3	?	Proteostasis ^F^	deUb ^G^	Ub	[96]
PRMT5	N		diMeR361/586		[97]
UBC9 ^H^	N	?	SUMO K111		[98]
E2/E3 Ub complex ^I^	?	?	Ub		[96]

^A^ PMT: Post-translational modification; ^B^ ↑: Stimulate interaction; ^C^ ↓: Prevents interaction; ^D^ Ku70/80/DNAPkcs is a DNA–PK complex; ^E^ K?PAR: PARylation at unknown lysine residues; ?: Unknown; ^F^ Proteostasis: Physiological regulation of Tdp1 levels: ^G^ deUb: De-ubiquitinylation; ^H^ UBC9: Indirect conclusion as Tdp1 is SUMOylated at K^111^ and this is the sole SUMO conjugating E2 enzyme, which could be facilitated by an unidentified SUMO E3 ligase protein; ^I^ E2/E3 Ub complex: Indirect conclusion as Tdp1 is Ubiquitylated at unknown Lys residues by an unidentified couple of Ubiquitin E2 conjugation/E3 ligase enzymes.
